# Quantitative evaluation of hindlimb grip strength in mice as a measure of neuromuscular function

**DOI:** 10.1016/j.mex.2024.103118

**Published:** 2024-12-25

**Authors:** Yaochao Zheng, Alexander Lunn, Jinghui Gao, Hongyu Chen, Yao Yao

**Affiliations:** Regenerative Bioscience Center, Department of Animal and Dairy Science, College of Agricultural and Environmental Science, University of Georgia, Athens, GA 30602, United States

**Keywords:** Grip strength, Mouse behavioral tests, Neuromuscular disorders, Single-hindlimb / Two-hindlimbs grip strength test

## Abstract

Muscle strength is a crucial metric for assessing motor function, with significant diagnostic and prognostic value. It is widely used in clinical and preclinical studies as a phenotypic indicator. In mouse models of neuromuscular disorders, grip strength provides a direct, repeatable measure of motor function changes throughout disease progression. Hindlimbs are critical evaluative targets in research due to their relevancy to rodent motor functions, but assessing their strength remains a challenge. Existing methods, such as the wire-hanging test, *in vivo* quadriceps contractility measurements, and isolated muscle or myofiber tests, each have limitations. The wire-hanging test, though repeatable, does not explicitly isolate hindlimbs, while *in vivo* contractility testing requires deep anesthesia, potentially compromising accuracy. Isolated muscle tests offer precise measurements but necessitate animal sacrifice, preventing longitudinal measurements. This study introduces an optimized method for assessing hindlimb grip strength that improves consistency and accessibility.

• It can be applied to measure both hindlimbs simultaneously, allowing for repeatable pre- and post-treatment comparisons.
• It enables single-hindlimb evaluation, supporting self-comparisons.
• This method is sensitive, user-friendly, and suitable for researchers of all expertise levels. It offers a robust tool for future research on neuromuscular interventions.

## Graphical abstract

**Figure:**
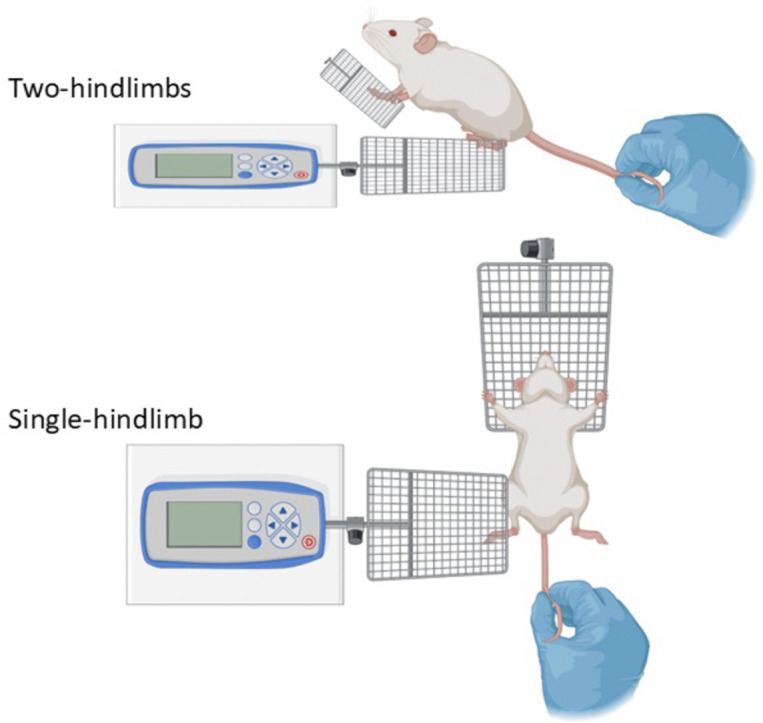

Specifications table

**Table:** 

Subject area:	Biochemistry, Genetics and Molecular Biology		
More specific subject area:	Muscle Biology		
Name of your method:	Single-hindlimb / Two-hindlimbs grip strength test		
Name and reference of original method:	N/A		
Resource availability:	**Name**	**Company**	**Catalog #**
	Chatillon DFS3–002 Digital Force gauge	Chatillon	
	Triangle ``trapeze'' bar	Columbus Instruments	0167–007M
	Mesh pull bar	Columbus Instruments	0167–007M
	Grip strength meter stand	Columbus Instruments	0167–007M

## Background

Skeletal muscle strength, the force a muscle can produce with a single maximal effort, is a crucial indicator of health and physical fitness [1]. It also serves as a functional determinant of adverse outcomes such as morbidity, disability, and mortality [2], declining with age or the progression of neuromuscular disease. Prior studies on individuals with neuromuscular disorders [3] found that changes in grip strength align closely with trends observed in other muscle strength tests, such as the 5-meter walk test, indicating that grip force measurements serve as a reliable indicator of overall muscle strength. Quantitative methods, such as isokinetic and portable dynamometers, offer reliable and valid measures of muscle strength in human patients [4,5].

Muscle strength measurement in rodent models has gained increased attention with the development of mouse models of progressive neuromuscular diseases. In rodent models, skeletal muscle force production can be measured in isolated whole-muscle or single myofiber preparations. The methods for force measurement in isolated skeletal muscles were delineated in the early-mid 20th century [6] and remain widely used in determining rodent motor defects. Limb muscles, such as extensor digitorum longus (EDL) and soleus (SOL), are typically used for contractility measurements, attributed to their accessibility and distinct fiber-type characteristics. While this method provides precise and detailed insights into muscle strength and excitation-contraction coupling, even at the single-fiber level, it requires muscle excision, necessitating sacrifice of the mice, thus making self-comparison (e.g., pre-treatment versus post-treatment) and longitudinal measurement impossible. Several methods have been developed and optimized for repeated and non-invasive assessments of muscle strength, including wire hanging test, *in vivo* quadriceps muscle contractility measurement [7], and isometric peak tetanic torque of the knee extensors [8] and plantar flexor muscle [9]. However, these methods have notable limitations. For instance, the wire-hanging test involves all four limbs, making it challenging to assess hindlimb strength specifically. On the other hand, *in vivo* muscle contractility measurements and isometric tetanic torque require placing mice under deep anesthesia, which may cause unpredictable side effects and potentially alter their behaviors. Due to these limitations, obtaining data that accurately represents muscle strength without sacrificing accuracy or repeatability poses a challenge.

The technique outlined in this paper addresses these challenges by integrating the precision and sensitivity of digital grip strength meters with an optimized handling approach and a specialized grasping apparatus. This method provides a safe and reliable alternative for hindlimb muscle strength assessment in mice, enabling repeatable and consistent measurements suitable for longitudinal studies. Importantly, it allows for the isolation of individual limbs, facilitating within-subject comparisons of muscle function and expanding possibilities for targeted muscle strength testing in rodent models.

## Method details

Set up digital force gauges.

1. Use gloves and other necessary personal protective equipment (PPE) for all following steps to avoid introducing contaminants.
2. Place the digital force gauge on a level surface within a designated small animal procedure room that meets Specific Pathogen-Free (SPF) standard (Fig. 1A). Ensure that the room meets all SPF standards, including controlled air filtration, humidity, and temperature, to maintain sterility and reduce contamination risks.

**Fig. 1:**
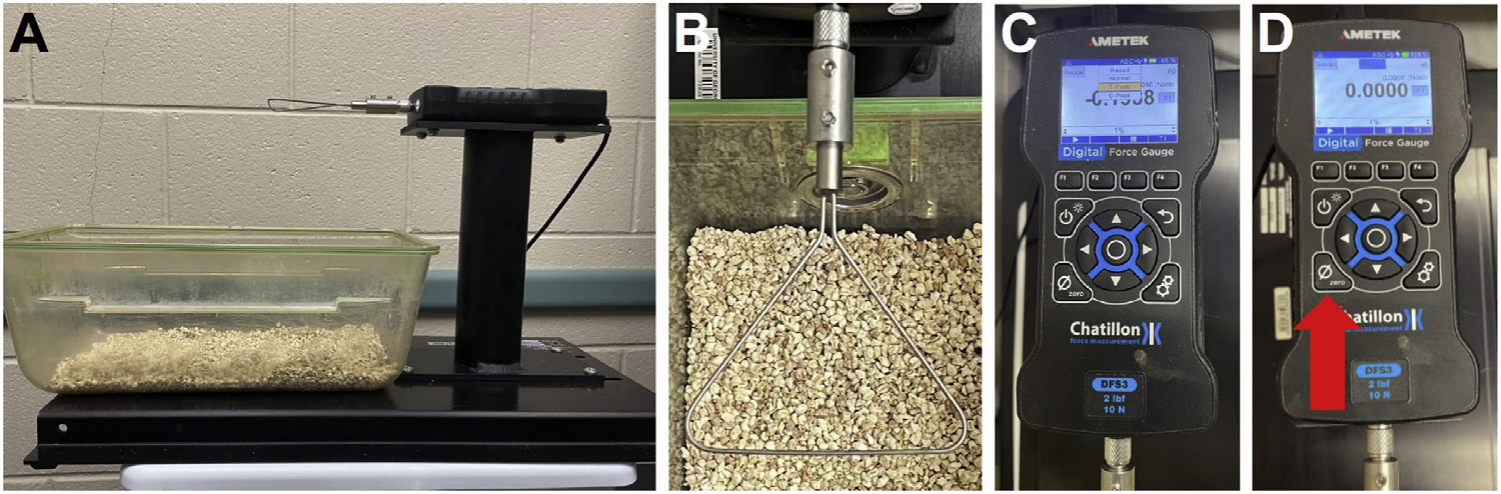
Set up of the digital force gauge. **(A)** Overview of the digital force gauge setup, including the Chatillon DFS3 force gauge and mouse container, placed on a level surface. **(B)** A triangular “trapeze” bar attachment is connected to the digital force gauge. **(C)** Location of the “T-Peak” mode on the force gauge. **(D)** Location of the zeroing feature button on the force gauge.
3. Adjust the gauge's height according to the operator's preference, keeping the sterile environment during setup.
4. Attach the triangular “trapeze” bar to the gauge (Fig. 1B).
5. Choose the “T-Peak” mode on the gauge (Fig. 1C) and set the reading to zero before testing the limbs (Fig. 1D).
6. Prepare the recording sheet.

### Measurement of combined grip strength in both hindlimbs

1. Hold the mouse by the mid-tail during the whole testing process (Fig. 2A).

**Fig. 2:**
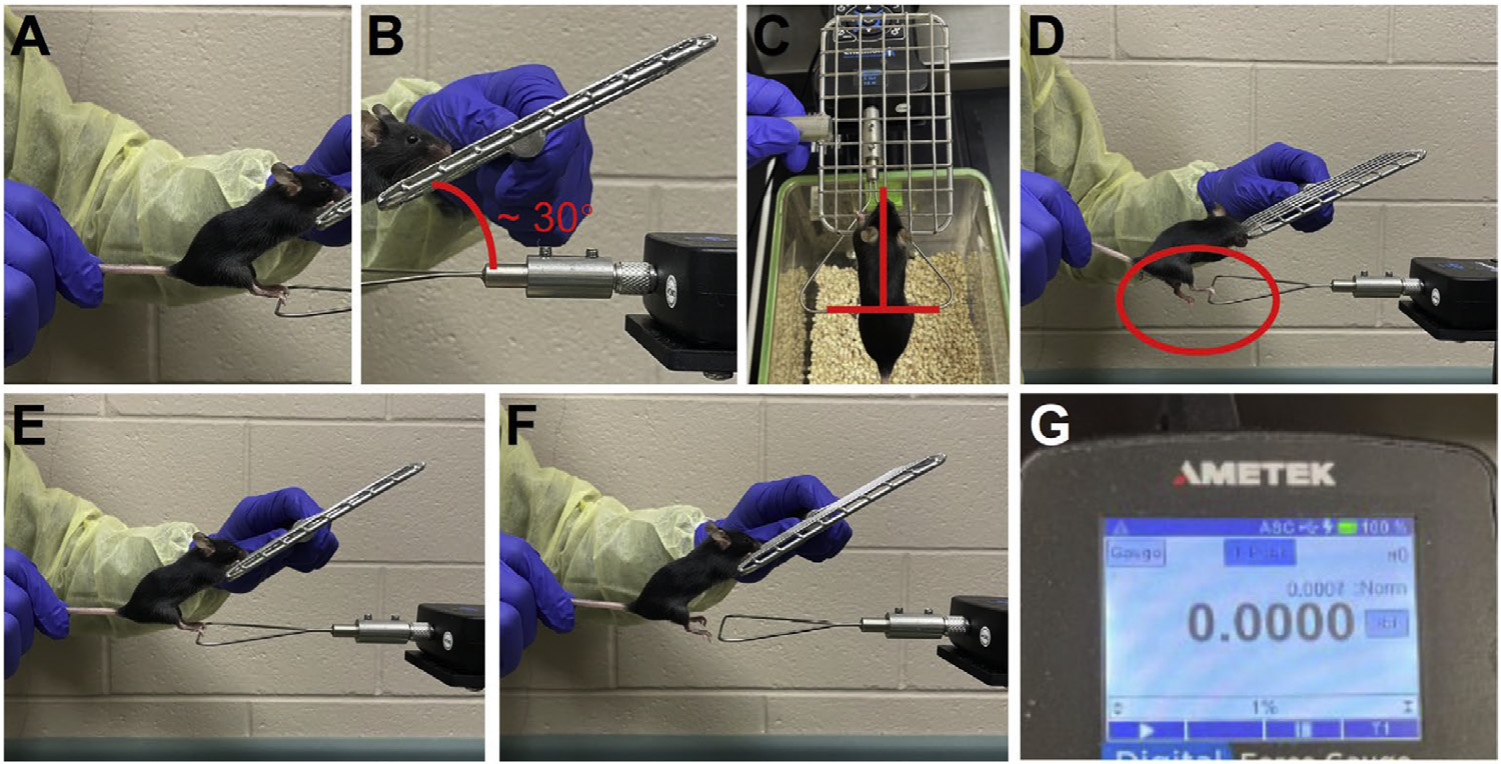
Measurement of combined two-hindlimbs grip strength. **(A)** Proper tail handling for securing the mouse. **(B)** The mesh pull bar is set at a 30 ° upward angle relative to the attached frame. **(C)** Correct placement of the mice's forelimbs on the mesh pull bar and the hindlimbs on the horizontal bar of the attachment frame. **(D)** Incorrect placement of only one hindlimb on the horizontal bar. **(E)** Apply a gentle, horizontal force to prompt a pulling action. **(F)** Coordinated pulling of the mouse and horizontal movement of the mesh pull bar until hindlimbs disengage from the bar. **(G)** Reading of grip strength on gauge.
2. Hold the mesh pull bar with the other hand and place it above the gauge frame at a 30 ° angle, leaning back from the operator (Fig. 2B).
3. Place only the forelimbs on the closer-end mesh.
4. Adjust the mouse and the mesh pull bar simultaneously and slowly until the mouse can rest its hindlimbs on the horizontal bar of the frame. Ensure the mouse torso is kept vertical to the bar (Fig. 2C) and both legs are holding the bar. Prevent any application of this technique with only one leg (Fig. 2D).
5. Apply a slight force and observe the hindlimbs to ensure the mouse holds the bar firmly (Fig. 2E).
6. Pull the mouse horizontally from the gauge until its hindlimbs come off the bar (Fig. 2F). Move the mesh pull bar at the same speed and direction as the hand holding the mouse.
7. Record the reading on the gauge (Fig. 2G).
8. Reset the reading to zero before repeating.

### Measurement of grip strength of single hindlimb

1. Hold the mouse by the mid-tail during the whole testing process.
2. Hold out a mesh pull bar with the other hand. Place it between the gauge's frame and the operator and on the opposite side of the mouse leg to be tested (Figs. 3A and B). For example, when testing the left leg of the mouse, place the mesh pull bar on the right side. The mesh pull bar should remain parallel to the frame.

**Fig. 3:**
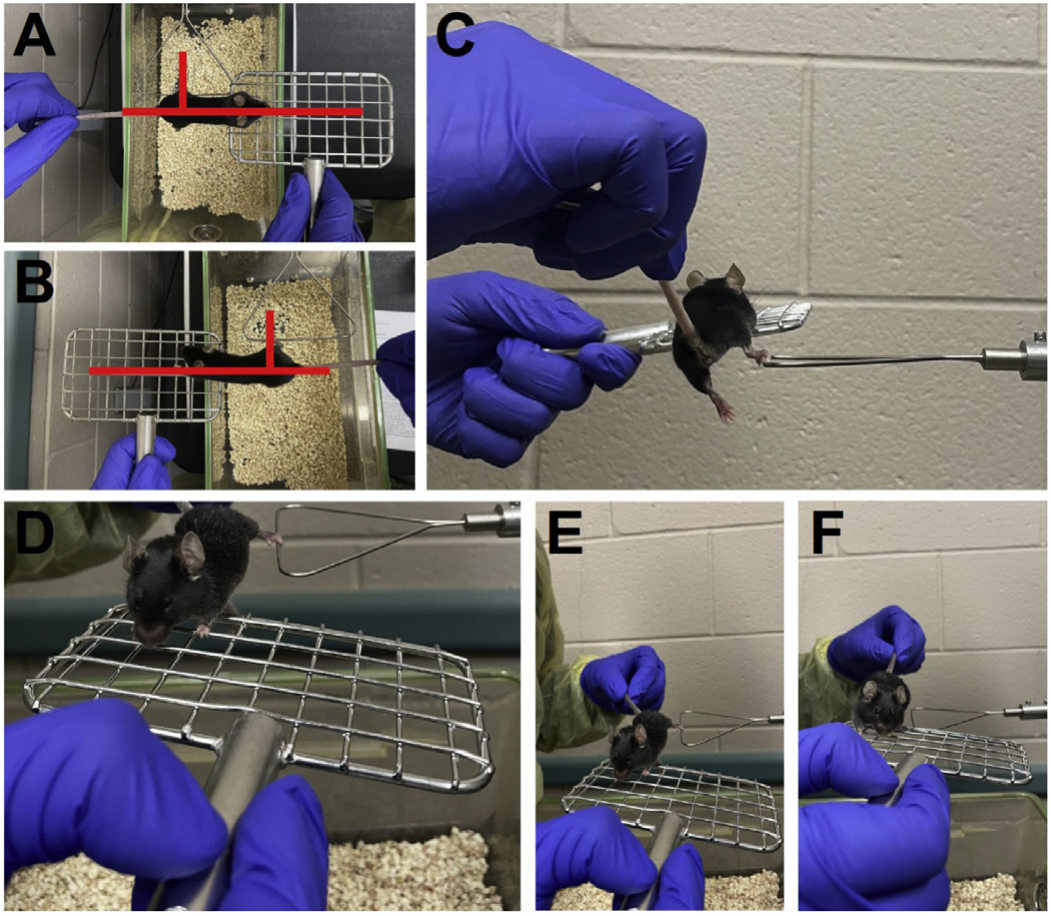
Measurement of single-hindlimb grip strength. **(A)** Proper placement of the mesh pull bar parallel to the gauge frame to test the left hindlimb. **(B)** Align the mesh pull bar parallel to the gauge frame to test grip strength of the right hindlimb. **(C)** Correct placement of mice forelimbs on the mesh pull bar and a singular hindlimb on the horizontal bar of the attachment frame. **(D)** Apply a gentle, horizontal pulling force on the mice. **(E)** Simultaneously pull the mouse and move the mesh pull bar parallel to the digital force gauge. **(F)** Separation of hindlimb from the triangular “trapeze” bar.
3. Place only the forelimbs on the side of the mesh pull bar (Fig. 3C). Adjust the mouse and the mesh pull bar simultaneously and slowly until the mouse can rest its hindlimb to be tested on the horizontal bar of the frame.
4. Apply a slight force and observe the hindlimb to firmly ensure the mouse holds the bar (Fig. 3D).
5. Continuously pull the mouse horizontally from the gauge until its hindlimb comes off the bar (Figs. 3E and F). Move the mesh pull bar at the same speed and direction as the hand holding the mouse.
6. Record the reading on the gauge. Reset the reading to zero before repeating.

### Data acquisition and analysis

1. While taking measurements, the operator should also observe the reading to ensure the final reading of maximum force appears immediately after the mouse lets go of the bar to prevent inaccurate or invalid readings.
2. Record the mouse's body weight daily after measuring grip strength.
3. Evaluate grip strength by the following formula:
  $$Grip\;strength\;\left(N/g\right)=\;\frac{Mean\;digital\;read\;\left(N\right)}{Body\;weight\;\left(g\right)}$$

## Method validation

**Fig. 4:**
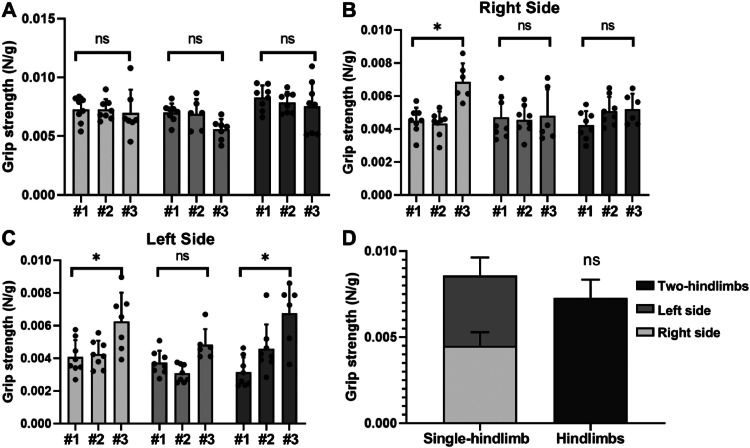
Representative measurements of combined and single-hindlimb grip strength. **(A)** Measurement of combined two-hindlimb grip strength by three different operators (#1, #2, and #3) for three mice. The results of each mouse are represented by a unique color. A total of 7 measurements per mouse were taken by each operator. **(B, C)** Measurement of single-hindlimb grip strength for the right leg (B) and left leg (C), again performed by three operators (#1, #2, and #3) on three mice, with 7 measurements per mouse taken by each operator. **(D)** The sum of the two single-hindlimb grip strengths was slightly higher than the combined two-hindlimb grip strength, although, this difference was not statistically significant (p > 0.05). NS, not significant. * P < 0.05.

Using methods described in this study, three trained operators measured the combined grip strength of two-hindlimbs in wild-type adult mice (n = 3). As shown in Fig. 4A, there is no significant difference in combined two-hindlimbs grip strength among operators (p > 0.05). Next, we measured single-hindlimb grip strength. The difference in single-hindlimb grip strength of the same mouse was evident among operators, especially for the left hindlimb. Furthermore, we found that the sum of two single-hindlimb grip strengths is slightly higher than the combined hindlimb grip strength (p > 0.05).

## Limitations

It is not recommended to repeat measurements on the same mouse too frequently within a short period, as the results may be inaccurate if the mice are nervous and agitated. This might explain why the standard deviation for the last operator is slightly larger. Additionally, the effectiveness of this technique may depend on the operator's familiarity with the protocols, as familiarity with the mice allows the operator to obtain more consistent and reliable data. To avoid technical bias, it is essential to have the same individuals perform the grip strength assessments throughout the entire project. This can significantly reduce artificial interruptions and better reflect the mouse's performance and trends. In this study, the variance in the left leg is slightly more significant than in the right leg, possibly due to the operator's handedness.

## Ethics statements

Adult male wild-type (C57BL/6J, stock #000664) mice were purchased from the Jackson Laboratory and used in this study. The use of animals was approved by the Institutional Animal Care and Use Committee at the University of Georgia. The studies complied with the National Institutes of Health Guidelines for the care and use of animals in research. The animals were maintained in the animal facilities in the Department of Animal and Dairy Science at the University of Georgia.

## CRediT author statement

**Yaochao Zheng:** Conceptualization, Writing-Original draft preparation. **Alexander Lunn:** Methodology, Validity tests. **Jinghui Gao:** Validity tests, Statistical analysis. **Hongyu Chen:** Methodology, Validity tests. **Yao Yao:** Conceptualization, Supervision, and Writing-Reviewing and Editing.

## Declaration of competing interest

The authors declare that they have no known competing financial interests or personal relationships that could have appeared to influence the work reported in this paper.

## Data Availability

No data was used for the research described in the article.
